# Characterization of protein adsorption onto silica nanoparticles: influence of pH and ionic strength

**DOI:** 10.1007/s00396-015-3754-x

**Published:** 2015-09-11

**Authors:** Jens Meissner, Albert Prause, Bhuvnesh Bharti, Gerhard H. Findenegg

**Affiliations:** Stranski Laboratorium, Institut für Chemie, Technische Universität Berlin, Straße des 17. Juni 124, 10623 Berlin, Germany; Department of Chemical & Biomolecular Engineering, NC State University, Raleigh, NC 27695 USA

**Keywords:** Adsorption, Protein, Nanoparticles, Lysozyme, ß-lactoglobulin, Silica, Electrostatic interactions

## Abstract

The adsorption of lysozyme and ß-lactoglobulin onto silica nanoparticles (diameter 21 nm) was studied in the pH range 2–11 at three different ionic strengths. Since the two proteins have a widely different isoelectric point (pI), electrostatic interactions with the negative silica surface lead to a different dependence of adsorption on pH. For lysozyme (pI ≈ 11), the adsorption level increases with pH and reaches a value corresponding to about two close-packed monolayers at pH = pI. In the multilayer adsorption region near pI, added electrolyte causes a decrease in adsorption, which is attributed to the screening of attractive interactions between protein molecules in the first and second adsorbed layer. For ß-lactoglobulin (pI ≈ 5), a pronounced maximum of the adsorbed amount is found at pH 4 in the absence of salt. It is attributed to the adsorption of oligomers of the protein that exist in the solution at this pH. An inversion in the influence of salt on the adsorbed amount occurs at pH > pI, where the protein and the surface are both negatively charged. This inversion is attributed to the screening of the repulsive protein-surface and protein–protein interactions. The adsorption isotherms were analyzed with the Guggenheim–Anderson–De Boer (GAB) model, which allows for two adsorption states (strongly and weakly bound protein).

## 1. Introduction

Globular proteins are strongly adsorbed to hydrophobic as well as hydrophilic interfaces due to the patchwise hydrophobic/hydrophilic character of their surface. The “multipolar” nature of proteins—as distinct from “bipolar” surfactants—leads to specific phenomena in the adsorption onto nanoparticles and emulsion droplets [1]; whereas surfactants cause a stabilization of dispersions and emulsions, adsorption of proteins makes the particles/droplets “sticky,” when attractive patches exist on opposite sides of the protein molecule. In such cases, the adsorption of the protein can cause bridging aggregation and flocculation of the particles [1–4]. This paper presents a study of protein adsorption onto silica nanoparticles under the influence of this protein-induced flocculation.

The interaction of proteins with nanoparticles (NPs) plays an important role in biotechnology and biomedical applications. In a biological environment, the NPs are exposed to a variety of proteins which may or may not be adsorbed to the particle surface, depending on the strength of protein-particle interaction [5]. In the past decade, many aspects of protein interaction with NPs have been investigated [6]. It has been found that the strength of protein–surface interaction and the secondary structure of adsorbed proteins is affected by the NP size [7–10] and the hydrophilic or hydrophobic nature of the NPs [10–13]. Electrostatic interactions play an important role in the adsorption of proteins at hydrophilic/charged surfaces. It is thought that conformationally stable (“hard”) proteins are adsorbed at charged surfaces only under electrostatically attractive conditions [14]. Specifically, at a negatively charged surface, only proteins with a net positive charge should be adsorbed, i.e., proteins having an isoelectric point pI higher than the pH of the solution. However, recent studies of protein adsorption into polyelectrolyte brushes have shown that a protein can be strongly adsorbed into a brush having the same charge as the protein, i.e., adsorption takes place at the “wrong side” of its isoelectric point [15–17]. It was proposed that this is a consequence of the “patchy” charge distribution on the protein surface, which implies that a protein of net negative charge can still have patches of positive charge. When a protein near the surface is oriented such that a positive patch points toward the negatively charged surface, an attractive interaction of entropic origin can arise as a result of the release of counter-ions [15, 18]. In fact, it has long been recognized that the binding strength of a protein is determined by a small number of charged groups located in the contact region on the surface of the protein [19]. An alternative explanation for protein adsorption at the wrong side of the isoelectric point is based on the charge regulation effect. Since the ionizable groups on the protein represent weak acids and bases, their charge is dependent on pH, and thus, their degree of dissociation will be influenced by the local electrostatic field near the surface. Next to a negatively charged surface, the pH is lower and the protein charge more positive than in the bulk solution [20, 21].

The role of electrostatic interactions in protein adsorption onto silica and metal oxide surfaces has been considered in many studies [22–25]. Commonly, it is found that the adsorbed amount as a function of pH reaches a maximum near pI of the protein [22, 24]. Since the net charge is zero at pI, the electrostatic repulsion between adsorbed protein molecules is at a minimum, and thus, the molecules can attain a closer packing at the surface than when carrying a net charge. Van der Veen et al. [24] performed a comparative adsorption study of two proteins of different pI at a macroscopic silica surface. It was found that added electrolyte affects the protein adsorption at the two sides of pI in opposite ways, which indicates the importance of electrostatic protein–protein and protein–surface interactions. Here, we present a similar comparative study for the adsorption of proteins at silica NPs. In this case, the adsorption behavior may also be affected by the surface curvature and the protein-induced aggregation of the particles, which in turn is also dependent on pH and ionic strength [2, 3]. Protein adsorption onto NPs can be determined either by measuring the depletion of the solution after equilibration with the NPs, or indirectly from the increase in size of the NPs due to the formation of a protein layer. The latter method avoids errors in the measurement of protein concentration, but it is indirect, as it relies on a suitable adsorption isotherm equation [26]. A variety of isotherm equations for protein adsorption have been discussed in the literature, from classical ligand-binding models developed in biochemistry [27, 28] to models derived from modern statistical mechanics [29]. Most of the models assume that adsorption is limited to some maximum level, usually a monolayer of protein molecules. Although this will be a reasonable assumption in many circumstances, weaker adsorption beyond a monolayer has also been reported, particularly in a pH range close to the isoelectric point of the protein [2].

Lysozyme (Lyz) and ß-lactoglobulin (ß-Lg) were chosen for this comparative adsorption study. The two proteins have similar size and molar mass but a widely different isoelectric point. Important characteristics of the two proteins are given in Table 1. Lyz is a conformationally stable (“hard”) protein due to 4 intramolecular disulfide bonds, and no significant association of the protein occurs at concentrations relevant in the present context. ß-Lg has only two intramolecular disulfide bonds and is less stable than Lyz toward partial unfolding. It represents a mixture of two generic variants (A and B) differing only in two positions along the chain [30]. Depending on pH, temperature, ionic strength, and concentration, ß-Lg is present in different oligomeric forms [31]. It was of interest to find out how these differences in surface charge distribution and aggregation behavior affect the adsorption of the two proteins at silica NPs.

**Table 1:** Characteristic parameters of the proteins

Protein	Dimensions (nm)	Molar weight *M*_*P*_ (kDa)	Isoelectric point pI
Lysozyme	3 × 3 × 4.5	14.3	11.1 [23]
ß-lactoglobulin	3.6 × 3.6 × 3.6	18.4	5.2 [30]

## 2. Materials and methods

### Materials

Ludox TMA colloidal silica (Sigma-Aldrich) was used as the adsorbent in this study. The Ludox dispersion was dialyzed for 5 days against DI water (water changed 3 times per day) to remove remaining salt. Its mean particle diameter *D* was 21 nm (determined by dynamic light scattering). Its specific surface area *a*_*s*_ was 128 m^2^/g (value from the manufacturer), in agreement with the geometric surface area derived from the particle diameter, *a*_geom_ = 6/*ρ*_*s*_*D* = 130 m^2^/g, based on a mass density of silica *ρ*_*s*_ of 2.20 g/cm^3^. The value of *a*_*s*_/*a*_geom_ = 1.02 indicates a low surface roughness of the particles [32]. The electrophoretic mobility of the Ludox particles was determined by electrophoretic light scattering of a 1 wt% dispersion as described elsewhere [2], using a Nano Zetasizer (Malvern Instruments, UK). Three measurements, each consisting of at least 50 runs, were performed for each sample.

Lysozyme from chicken egg white lyophilized powder (Sigma-Aldrich, ≥40,000 units/mg protein, lot SLBH9534V, purity ≥90 %) and ß-lactoglobulin from bovine milk (Sigma-Aldrich, lot SLBC4958V, purity ≥90 %) were used in this study.

### Protein adsorption measurements

The amount of protein adsorbed on the silica NPs was determined by measuring the depletion of the supernatant solution after equilibration of the sample. For each adsorption isotherm at given pH and salt concentration, a stock solution of buffer (10 mM formiate, MES, BICINE, or CAPS) was freshly prepared and adjusted to the desired pH with aqueous HCl or NaOH solution (1 M). Stock solutions of protein (10 mg/mL) and NaCl (250 mM) were then prepared in the buffer solution. A portion of dialyzed Ludox TMA dispersion (about 30 wt%) was diluted in a volume ratio 1:2 with buffer solution to obtain the Ludox TMA stock solution (about 10 wt%). The mass fraction of silica in this stock solution was checked gravimetrically for each adsorption isotherm. The three stock solutions (silica NPs, protein, and buffer) were then mixed in known proportions to arrive at eight different protein concentrations (0.5–5 mg/mL), three different NaCl concentrations (0, 25, and 100 mM), and a constant mass fraction of Ludox TMA (about 1 wt%). The samples were equilibrated for 20 h at 20 °C in closed vials using a thermo-mixer. After equilibration, the samples were centrifuged for 3 h at 15,000 rpm (21,000*g*) to separate the supernatant from the silica. The possibility of systematic errors caused by sedimentation of non-adsorbed protein during centrifugation was checked by determining sedimentation isotherms of ß-Lg in the absence of NPs but under otherwise the same conditions (protein concentration, pH, salt concentration and centrifugation time) as in the adsorption measurements. It was found that this error was negligibly small under the experimental conditions.

Protein concentration in the supernatant solution was determined by UV–vis spectrometry. Sample spectra were compared to a protein standard (1 mg/mL), prepared from the same protein stock solution, in the wavelength range 265–300 nm. The best-fit value of the concentration was obtained by minimizing the sum of square deviation in absorbance from the concentration standard in the chosen wavelength range [33]. The surface concentration *Γ* of adsorbed protein (mass per unit surface area) was calculated from the depletion of the supernatant solution by the relation

1 $$ \varGamma =\frac{V\left({c}_0-{c}_{eq}\right)}{m_s{a}_s} $$

where *V* is the volume of protein solution of initial mass concentration *c*_0_ and final concentration *c*_*eq*_ after equilibration with a mass *m*_*s*_ of silica of specific surface area *a*_*s*_. The adsorbed amount was also expressed by the number of protein molecules per silica particle,

2 $$ N=\varGamma \frac{N_A{\rho}_s{a}_s{D}^3\pi }{6{M}_P} $$

where *M*_*P*_ is the molar weight of the protein and *N*_*A*_ the Avogadro constant.

### Adsorption isotherm equation

The liquid-phase version of the Guggenheim–Anderson–De Boer (GAB) model was used to represent the protein adsorption data. Similar to the BET relation, the GAB multilayer gas adsorption model [34] assumes that the state of adsorbate molecules in the second and all higher adsorption layers is the same, but different from that in the first layer. A further assumption of the GAB model is that the state of adsorbed molecules in the second and higher layers is also different from the bulk liquid state. The liquid-phase version of the GAB model takes up the concept of two distinct adsorption states: There are *N*_*m*_ equivalent adsorption sites per unit area to which adsorbate molecules bind strongly, and each occupied site can accommodate successively further adsorbate molecules in a weaker sorption state. This three-parameter adsorption isotherm has the form [35]

3 $$ \varGamma ={\varGamma}_m\frac{K_S{c}_{eq}}{\left(1-{K}_L{c}_{eq}\right)\left(1+{K}_S{c}_{eq}-{K}_L{c}_{eq}\right)} $$

where *Γ*_*m*_ = *N*_*m*_*M*_*P*_/*N*_*A*_ is the surface concentration of strongly adsorbed protein, *K*_*S*_ is the adsorption constant for molecules in the strong adsorption state, and *K*_*L*_ the adsorption constant of the weak adsorption state. Equation 3 reduces to the Langmuir equation when *K*_*L*_ = 0, but it yields values of *Γ* greater than *Γ*_*m*_ at high concentrations *c*_*eq*_ when *K*_*L*_ > 0. The familiar BET equation for vapor adsorption is recovered from Eq. 3 by setting *K*_*L*_*c*_*eq*_ = *p*/*p*_0_ and introducing the parameter *C* = *K*_*S*_/*K*_*L*_.

## 3. Results

### Nanoparticle and protein characteristics

**Fig. 1:**
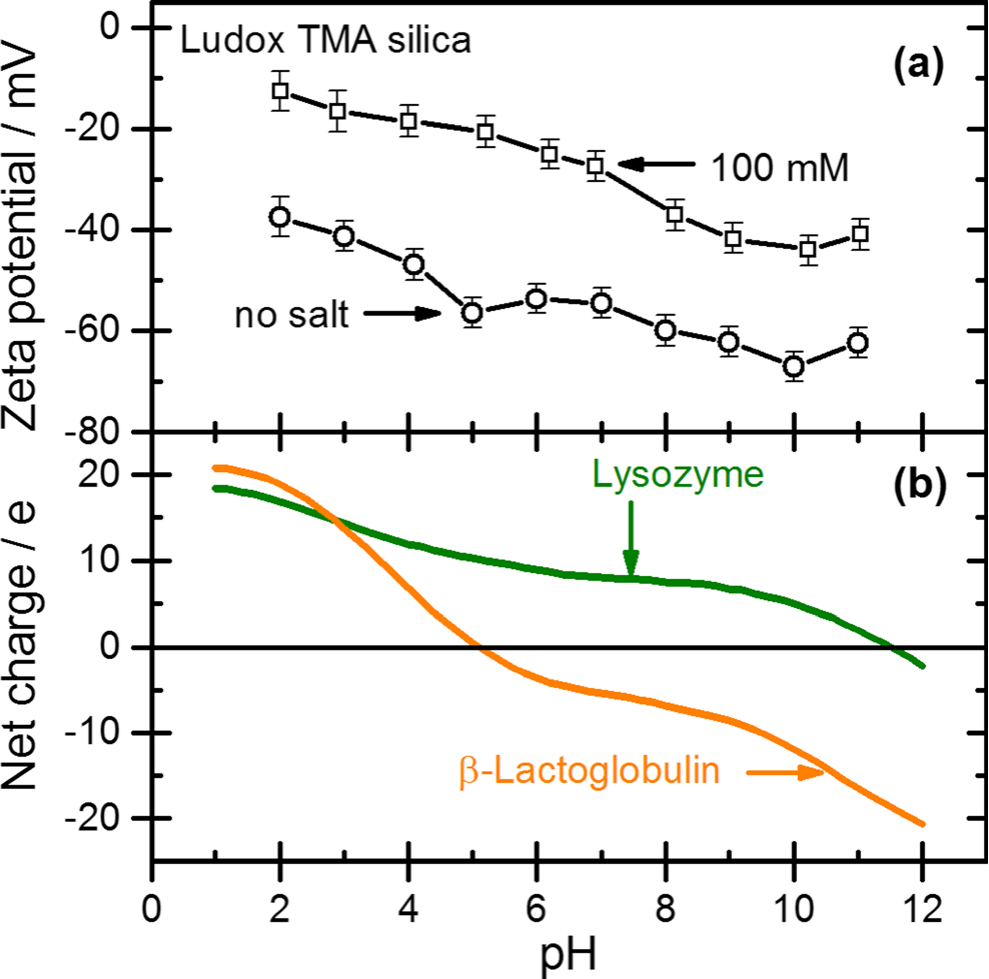
**a** Zeta potential of Ludox TMA silica particles as a function of pH for two different ionic strengths (see Table 2). **b** Estimated net charge of lysozyme and ß-lactoglobulin as a function of pH

The electrophoretic mobility *μ*_*e*_ of the Ludox TMA NPs was determined at several pH values in the absence of salt and in 100 mM NaCl, and the zeta potential *ζ* was calculated from the mobility by the Henry equation. The electrokinetic surface charge density *σ*_0_ of the particles was estimated from the zeta potential using the Gouy–Chapman relation (see ref. [36]). Results for *μ*_*e*_, *ζ*, and *σ*_0_ for several pH values are collected in Table 2. The dependence of the zeta potential on pH is shown in Fig. 1a. Note that the zeta potential of the Ludox TMA particles is negative in the entire pH range, i.e., no isoelectric point is observed down to pH 2.

**Table 2:** Electrophoretic mobility, zeta potential, and electrokinetic charge density of Ludox TMA silica nanoparticles as a function of pH without added salt and with 100 mM NaCl

Added salt	pH	Ionic strength	*μ*_*e*_	*ζ*	*σ*_0_
		mM	10^−8^ m^2^ s^−1^ V^−1^	mV	e nm^−2^
0 mM	2.0	10	−1.84	−37	−0.01
	3.0	1	−2.03	−41	−0.03
	4.1	7	−2.41	−47	−0.06
	5.0	1	−2.77	−57	−0.03
	6.0	4	−2.72	−54	−0.06
	7.0	9	−2.83	−55	−0.09
	8.0	3	−3.01	−60	−0.06
	9.0	8	−3.22	−62	−0.10
	10.0	3	−3.36	−67	−0.07
	11.0	8	−3.24	−63	−0.10
100 mM	2.0	110	−0.27	−13	−0.06
	2.9	101	−1.00	−17	−0.08
	4.0	106	−1.12	−19	−0.09
	5.2	101	−1.24	−21	−0.10
	6.2	106	−1.51	−25	−0.12
	6.9	109	−1.65	−27	−0.14
	8.2	104	−2.23	−37	−0.19
	9.1	108	−2.52	−42	−0.22
	10.2	104	−2.65	−44	−0.23
	11.0	108	−2.47	−41	−0.22

The net charge of the proteins was estimated from the numbers of the individual acidic and basic amino acids and their respective acidity constants [37]. The dependence of the estimated net charge on pH is shown in Fig. 1b. Both proteins have a high positive net charge at pH 2, but for ß-Lg, the net charge falls of steeply with increasing pH and becomes negative above pH 5.2 = pI. Lyz contains a larger number of basic amino acids than ß-Lg; hence, its net charge falls off less steeply, and its isoelectric point is reached only at pH 11. Accordingly, in the case of lysozyme the protein and the silica particles are oppositely charged from pH 2 to pH 11. In the case of ß-Lg the protein and silica particles are oppositely charged up to pH 5.2 but equally charged at higher pH.

### Lysozyme adsorption

**Fig. 2:**
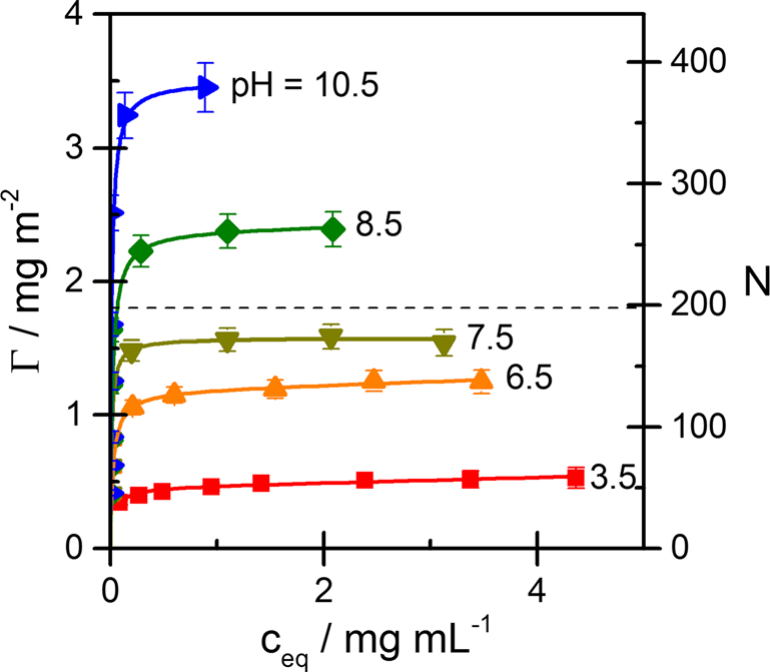
Adsorption isotherms of lysozyme on Ludox TMA for several pH values without added salt: Experimental data (symbols) and fits by the GAB model (lines). The adsorbed amount is expressed as protein mass per unit area (*Γ*) and by the mean number of protein molecules per silica particle (*N*). The monolayer capacity based on a dense packing of protein molecules in side-on orientation (*A*_0_ = 13.5 nm^2^) is indicated by the *dashed line*

The adsorption of Lyz on Ludox TMA silica NPs was studied in a pH range 3.5 to 11.2 in the absence of salt and in 25 and 100 mM NaCl solutions. Figure 2 shows adsorption isotherms (20 °C) for a series of pH values up to the isoelectric point in the absence of added salt. Adsorption is expressed by the surface concentration *Γ* (mg/m^2^) and by the average number of protein molecules per silica particle (*N*) and is plotted against the concentration *c*_*eq*_ of protein in the equilibrated solution. The isotherms are of high-affinity type, i.e., sharply increasing at low concentrations and leveling off at higher concentrations. The adsorption level attained in the experimental concentration range is below 0.5 mg/m^2^ at pH 3.5, but strongly increasing with pH to a value close to 4 mg/m^2^ at pH 11.2 (not shown in Fig. 2). From the cross-sectional area of Lyz adsorbed side-on (*A*_0_ ≈ 4.5 nm × 3 nm = 13.5 nm^2^), the monolayer capacity is about 1.8 mg/m^2^, as indicated by the dashed line in Fig. 2. It can be seen that this adsorption level is nearly reached at pH 7.5, but higher values are attained closer to pI. A higher monolayer capacity (about 2.6 mg/m^2^) would result from head-on adsorption of the Lyz molecules. However, molecular simulation studies indicate that side-on adsorption is the preferred orientation of Lyz on negatively charged silica surfaces [38, 39]. Hence, the results in Fig. 2 indicate that adsorption exceeding a monolayer of protein molecules occurs at pH > 8.

**Fig. 3:**
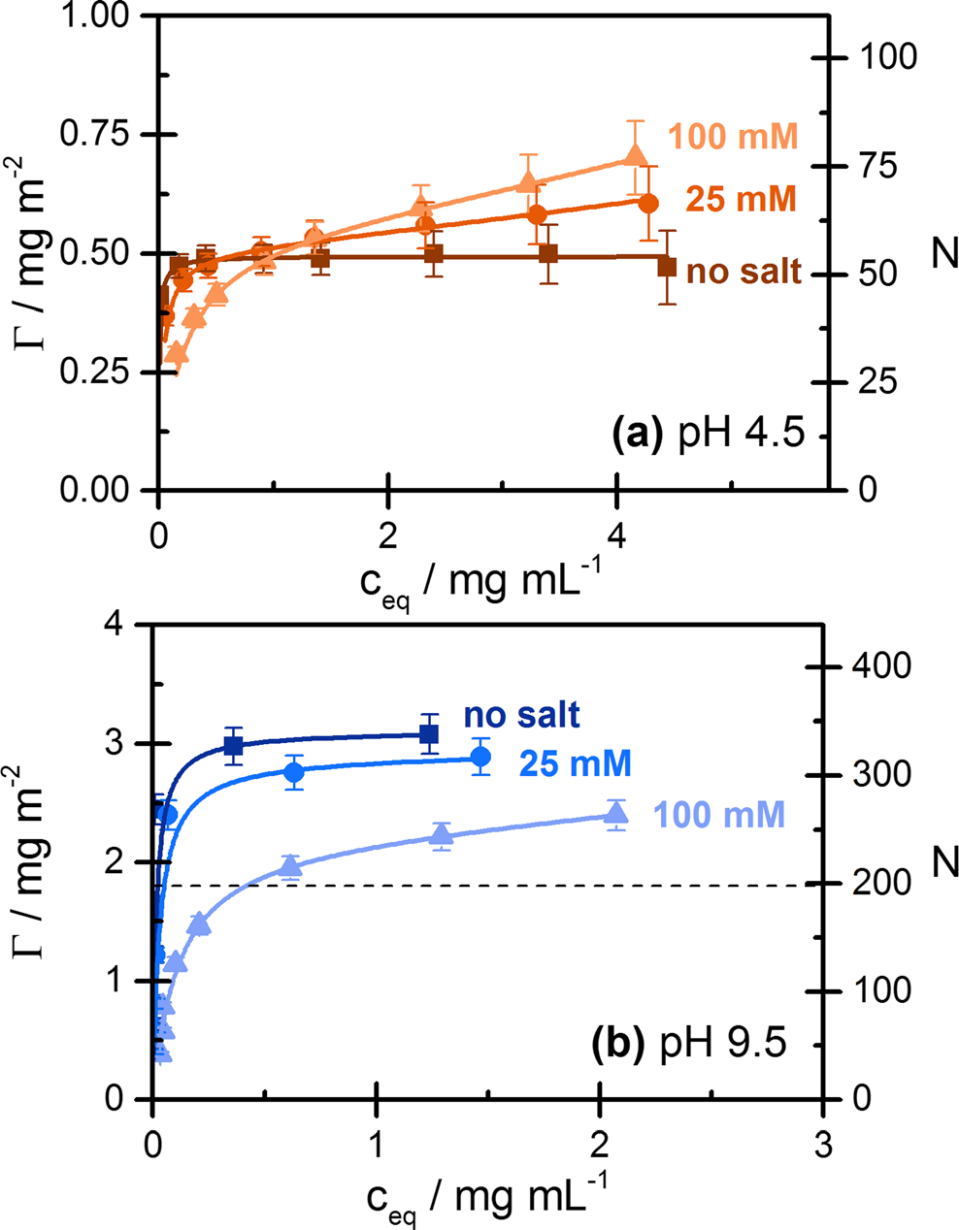
Adsorption isotherms of lysozyme on Ludox TMA: Influence of added salt at pH 4.5 (**a**) and pH 9.5 (**b**): *filled square*, no added salt; *filled circle*, 25 mM NaCl; *filled triangle*, 100 mM NaCl, and fits by the GAB model; see caption of Fig. 2 for details

Figure 3 illustrates the influence of added NaCl on the adsorption isotherm of Lyz at a low and a high pH. In both cases, the high-affinity character of adsorption isotherms is lost when salt is added, but the influence on the adsorption level at higher protein concentrations is different in the two pH regimes: At pH 4.5 (Fig. 3a), when the protein is highly charged, adsorption continues to increase in the presence of salt, becoming higher than the plateau value reached in the absence of salt. At pH 9.5 (Fig. 3b), when the adsorption level exceeds one nominal monolayer, added salt causes a significant decrease of the adsorption level at all protein concentrations studied in this work.

**Fig. 4:**
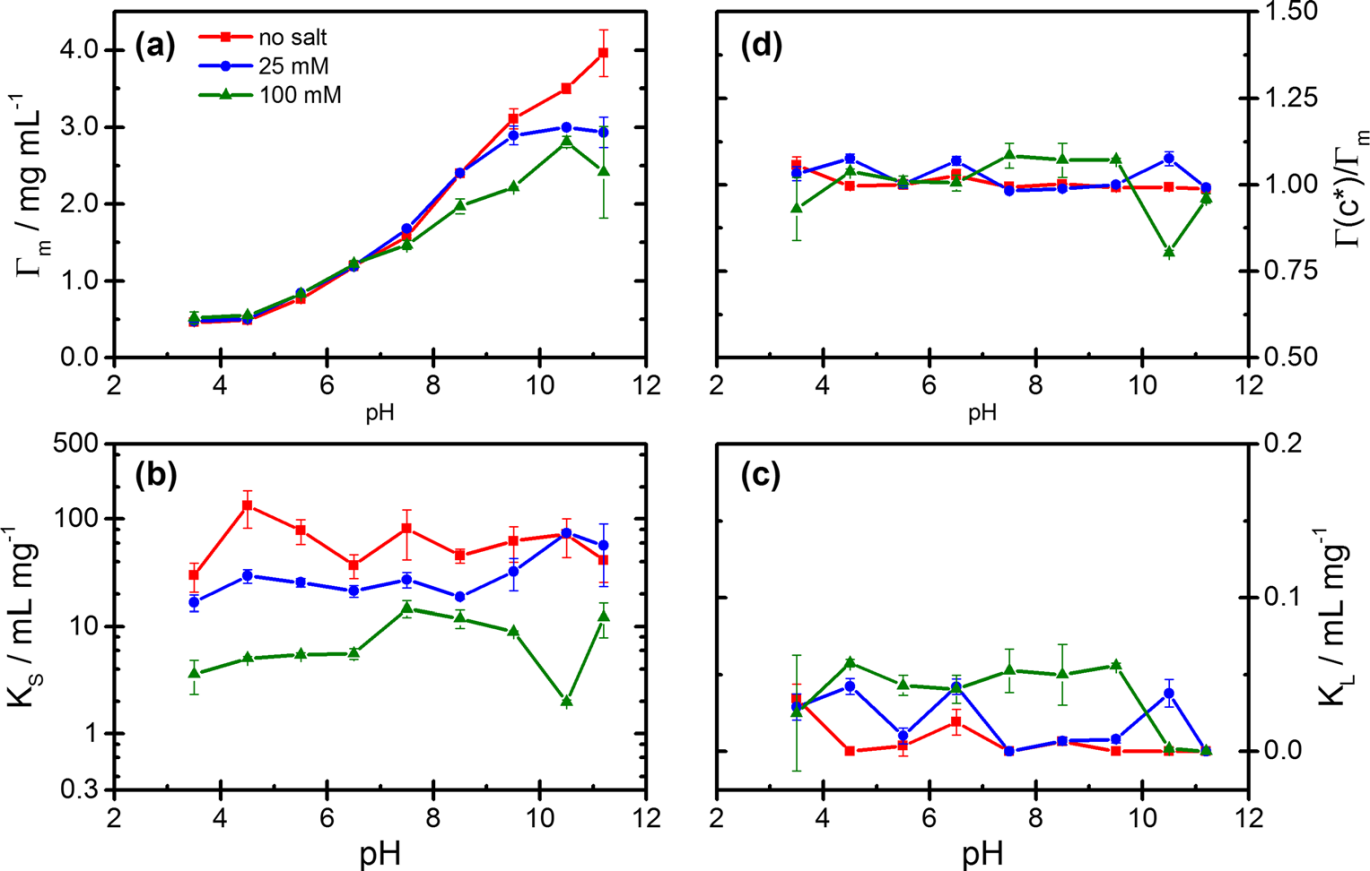
Lysozyme adsorption: Fit values of the GAB parameters as a function of pH for three ionic strengths (no salt, 25 mM, 100 mM NaCl): **a** limiting surface concentration *Γ*_*m*_ of strongly bound protein; **b** adsorption constant *K*_*S*_; **c** adsorption constant *K*_*L*_; **d** adsorption ratio *Γ*(*c* ^*^)/*Γ*_*m*_ for *c* ^*^ = 2 mg/mL (see text)

The experimental adsorption data can be represented by the GAB isotherm equation (Eq. 3), as shown by the full curves in Figs. 2 and 3. Values of the parameters *Γ*_*m*_, *K*_*S*_, and *K*_*L*_ obtained by fitting the adsorption data with Eq. 3 are presented as a function of pH in Fig. 4. The limiting surface concentration *Γ*_*m*_ of strongly adsorbed protein (Fig. 4a) is increasing with pH and reaches values above 3 mg/m^2^ near pI. Hence, in this pH region, the surface concentration of strongly bound protein clearly exceeds a monolayer of closely packed molecules. The adsorption constant *K*_*S*_ for the strongly bound protein (Fig. 4b), which relates to the high-affinity region of the adsorption isotherms, exhibits no systematic dependence on pH, but a systematic decrease with increasing salt concentration. The adsorption constant *K*_*L*_ of Lyz in the weakly bound state (Fig. 4c) is smaller by 2–3 orders of magnitude than *K*_*S*_. Like *K*_*S*_, it shows no systematic dependence on pH but some increase with the ionic strength. To quantify the contribution of the weak adsorption state to the overall adsorption, we introduce the adsorption ratio *Γ*(c^*^)/*Γ*_*m*_, where *Γ*(c^*^) represents the adsorbed amount at a reference concentration, c^*^ in the flat region of the isotherms as calculated by Eq. 3. Values of *Γ*(c^*^)/*Γ*_*m*_ <1 indicate that at the chosen reference concentration, the adsorbed amount is lower than the limiting concentration *Γ*_*m*_ of strongly adsorbed protein, while *Γ*(c^*^)/*Γ*_*m*_ > 1 implies that the weak adsorption state contributes to the overall adsorption. Figure 4d shows the adsorption ratio as a function of pH for a reference concentration c^*^ = 2 mg/mL. Values of *Γ*(c^*^)/*Γ*_*m*_ close to 1 are found in the absence of salt, indicating that in this case all adsorbed Lyz is strongly bound. At pH >7, where added salt causes a decrease of the adsorbed amount (Fig. 3), values of *Γ*(c^*^)/*Γ*_*m*_ >1 indicate that the salt-induced decrease of adsorption involves the participation of weak adsorption sites. These findings will be discussed in Section 4.

### ß-lactoglobulin adsorption

**Fig. 5:**
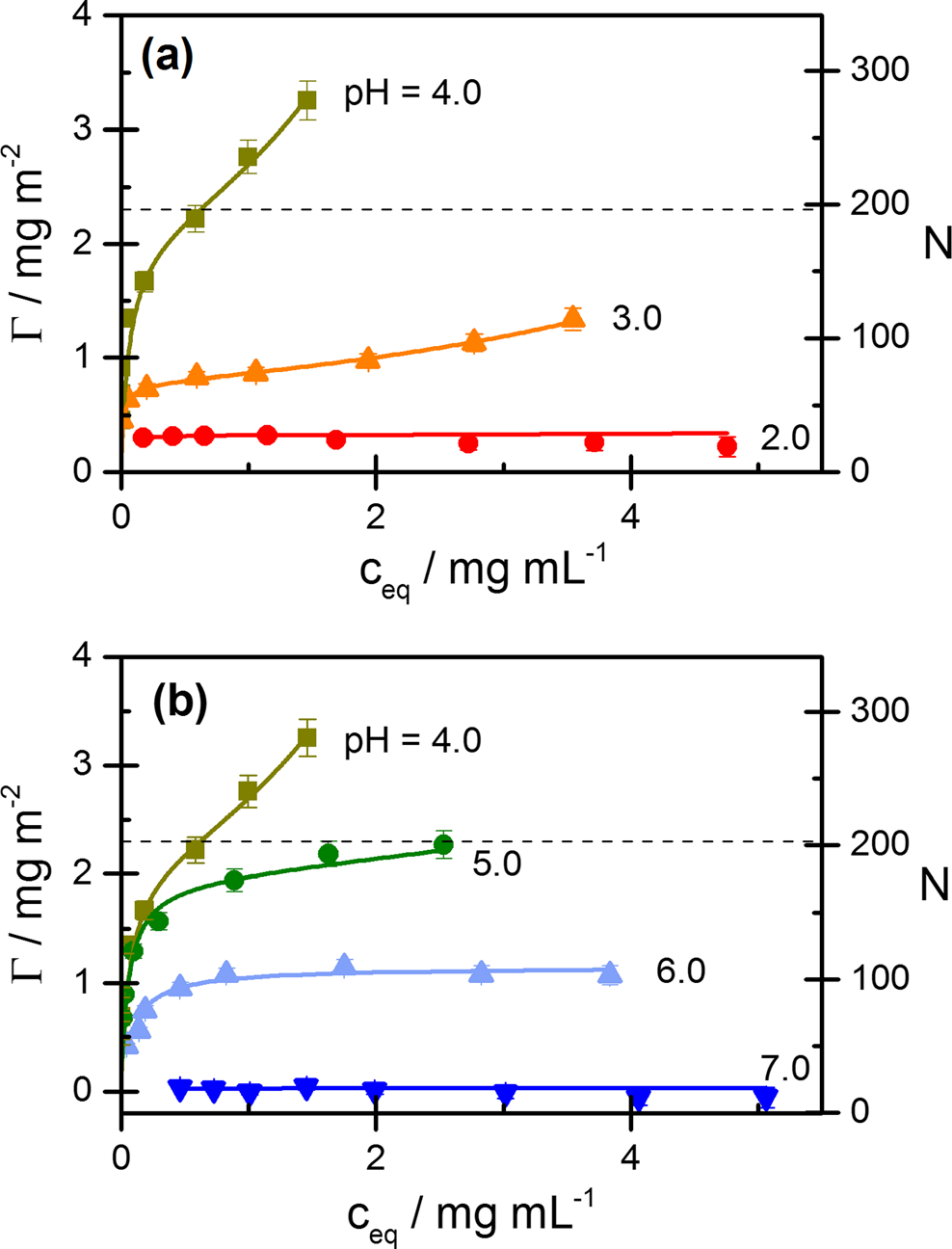
Adsorption isotherms of ß-lactoglobulin on Ludox TMA for several pH values without added salt: **a** pH ≤ pH 4; **b** pH ≥ pH 4. Experimental data (*symbols*) and fits by the GAB model (*lines*). The monolayer capacity based on a dense packing of monomeric protein (*A*_0_ = 13 nm^2^) is indicated by the *dashed line*; see also caption of Fig. 2

Adsorption isotherms of ß-Lg on Ludox TMA for a series of pH values in the absence of salt are shown in Fig. 5. It can be seen that adsorption sharply increases from pH 2 to pH 4 (Fig. 5a) and sharply decreases from pH 4 to pH 7 (Fig. 5b). All isotherms for pH <7 exhibit a high-affinity adsorption regime, even at pH 2, where the limiting adsorption is only 0.2 mg/m^2^. Beyond this high-affinity regime, a further increase of adsorption with protein concentration is observed at pH values near pI. This effect is most pronounced at pH 4. A monolayer of densely packed ß-Lg molecules (cross-sectional area *A*_0_ ≈ 3.6 nm × 3.6 nm ≈ 13 nm^2^) corresponds to a surface concentration of ca. 2.3 mg/m^2^ (dashed line in Fig. 5). This adsorption level is well exceeded at pH 4 but not at pH 5 (i.e., close to pI =5.2). At pH 6, when the net charge of the protein has changed from positive to weakly negative, there is still significant adsorption of the protein, but at pH 7 and higher, no adsorption of ß-Lg is detected in the absence of salt.

**Fig. 6:**
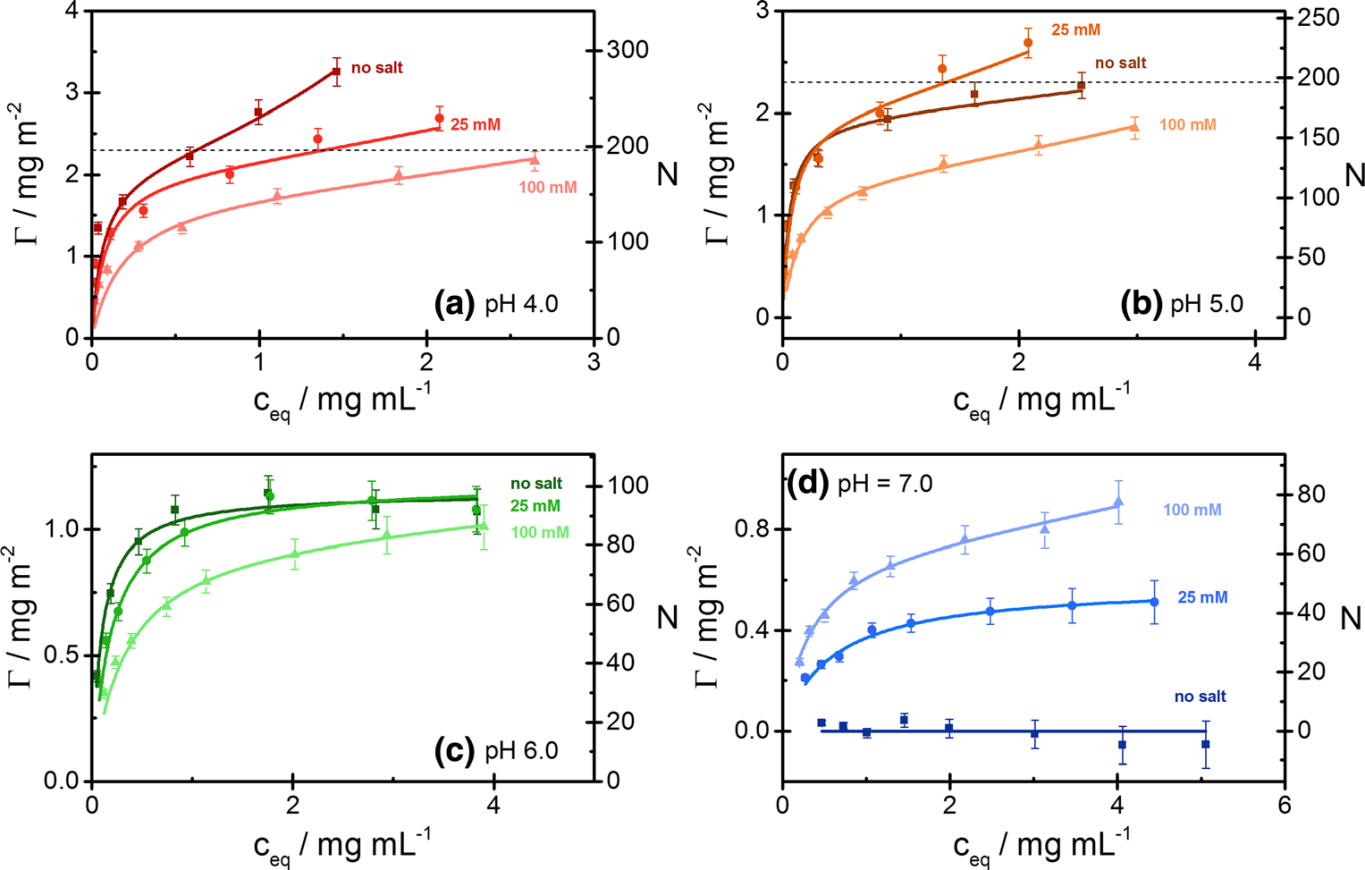
Adsorption isotherms of ß-lactoglobulin on Ludox TMA at **a** pH 4; **b** pH 5; **c** pH 6; **d** pH 7. Experimental data: *filled square*, no salt; *filled circle*, 25 mM NaCl; *filled triangle*, 100 mM NaCl; *full lines*: fit by the GAB equation. The estimated monolayer capacity is indicated by a *dashed line*. See caption to Fig. 2 for further details

The influence of salt on the adsorption of ß-Lg at different pH values is shown in Fig. 6, where the four panels demonstrate a reversal of the influence of salt on the protein adsorption in the range from pH 4 to 7: At pH 4 (Fig. 6a), the highest adsorption is found in the absence of salt and the lowest adsorption at 100 mM salt. Changing from pH 4 to pH 5 (Fig. 6b) causes a drastic decrease of adsorption in the absence of salt, but no significant decrease at 25 or 100 mM salt; as a consequence, the adsorption at no salt is now intermediate between that at 25 and 100 mM salt. Changing from pH 5 to pH 6 (Fig. 6c) causes further strong decrease in adsorption at no salt, and also a strong decrease at 25 mM salt, but again, no decrease of adsorption at 100 mM salt. Finally, at pH 7 (Fig. 6d), the adsorption in the absence of salt has fallen to zero, and the adsorption at 25 mM salt has fallen below the adsorption at 100 mM salt, thus completing the inversion of the protein adsorption level as a function of salt concentration.

**Fig. 7:**
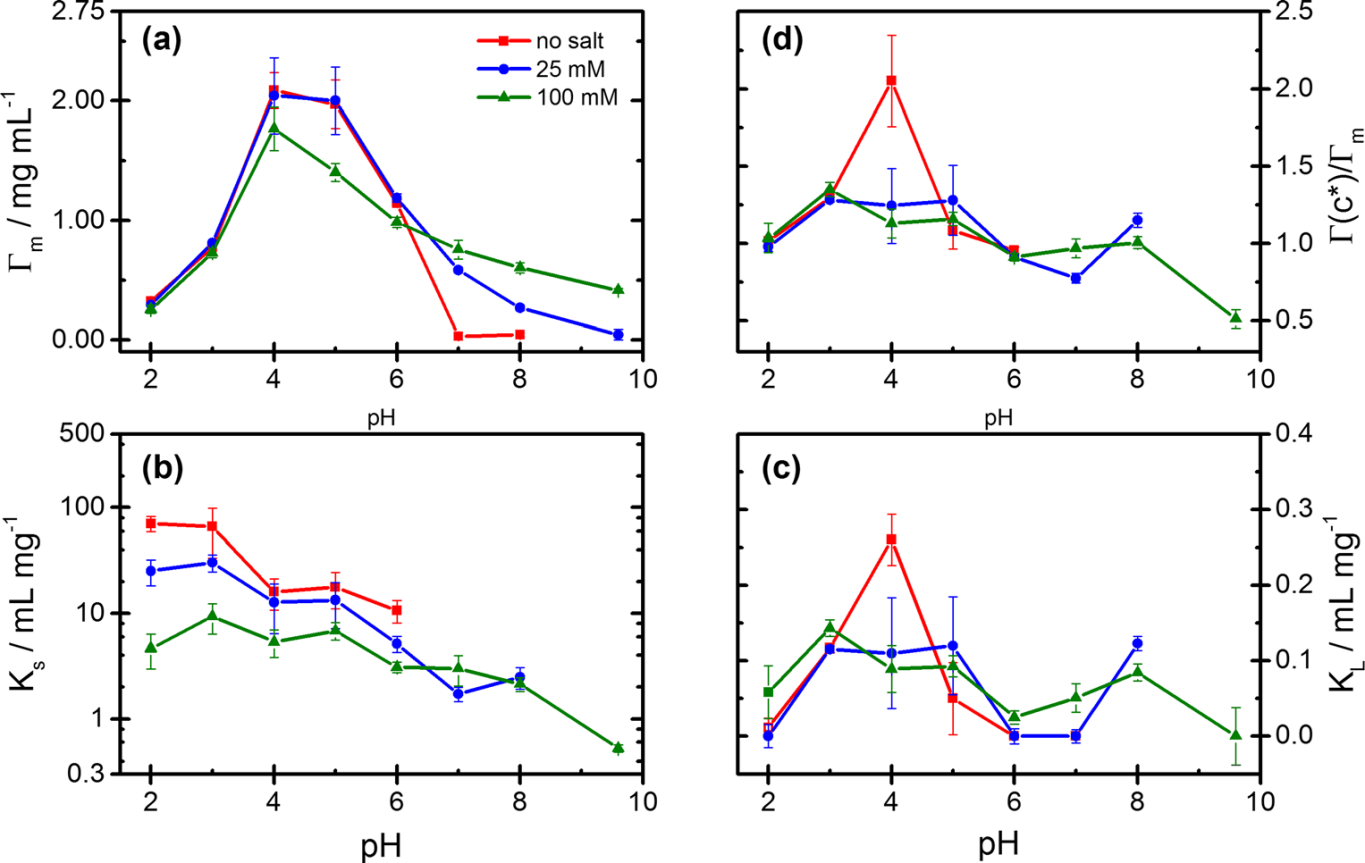
ß-Lactoglobulin adsorption: Fit values of the GAB parameters as a function of pH for three ionic strengths (no salt, 25 mM, 100 mM NaCl): **a** limiting surface concentration *Γ*_*m*_ of strongly bound protein; **b** adsorption constant *K*_*S*_; **c** adsorption constant *K*_*L*_; **d** adsorption ratio *Γ*(*c* ^*^)/*Γ*_*m*_ for *c* ^*^ = 2 mg/mL (see text)

The adsorption data for ß-Lg can again be represented by the GAB equation (Eq. 3), as shown by the full curves in Figs. 5 and 6. The parameters *Γ*_*m*_, *K*_*S*_, and *K*_*L*_ obtained from fits of the adsorption data and values of the adsorption ratio *Γ*(c^*^)/*Γ*_*m*_ at the reference concentration c^*^ = 2 mg/mL are shown as a function of pH in Fig. 7. The limiting surface concentration *Γ*_*m*_ of strongly bound protein (Fig. 7a) increases sharply at low pH, reaching a maximum at pH 4–5 and falls off more or less steeply at higher pH values, depending on the salt concentration. The highest values of *Γ*_*m*_, attained at pH 4–5 and low salt concentration correspond to nearly a monolayer of densely packed ß-Lg monomers (2.3 mg/m^2^). At pH above pI = 5.2, the values of *Γ*_*m*_ demonstrate the inversion of the influence of salt on the protein adsorption in this pH range. Unlike *Γ*_*m*_, the adsorption constant *K*_*S*_ of strongly bound protein (Fig. 7b) decreases in a monotonic way from low to high pH, without any singular behavior near pI. It also decreases with increasing salt concentration, though to a lesser extent than in the case of Lyz. Remarkably, values of *K*_*S*_ well above 1 mL/mg are still found in a pH range where both the surface and the protein are negatively charged. In contrast to *K*_*S*_, the adsorption constant *K*_*L*_ of ß-Lg in the weakly bound state (Fig. 7c) exhibits a pronounced maximum at pH 4 in the absence of salt, which disappears on addition of salt. In the presence of salt, *K*_*L*_ gradually decreases from pH 3 to pH 6 but appears to increase again at higher pH. The graphs of the adsorption ratio *Γ*(c^*^)/*Γ*_*m*_ for ß-Lg as a function of pH (Fig. 7d) again show the singular role of the weak adsorption state of ß-Lg at pH 4 in the absence of salt, where a pronounced maximum, *Γ*(c^*^)/*Γ*_*m*_ ≈ 2, is observed, implying that 50 % of the protein is adsorbed in the weakly bound state. Except for this singular point, *Γ*(c^*^)/*Γ*_*m*_ values in a range 1.1 to 1.3 are found for the pH range 3–5 and values close to 1 at higher pH. This indicates that protein in the weakly bound state plays a significant role in the neighborhood of the isoelectric point, but not elsewhere.

## 4. Discussion

**Fig. 8:**
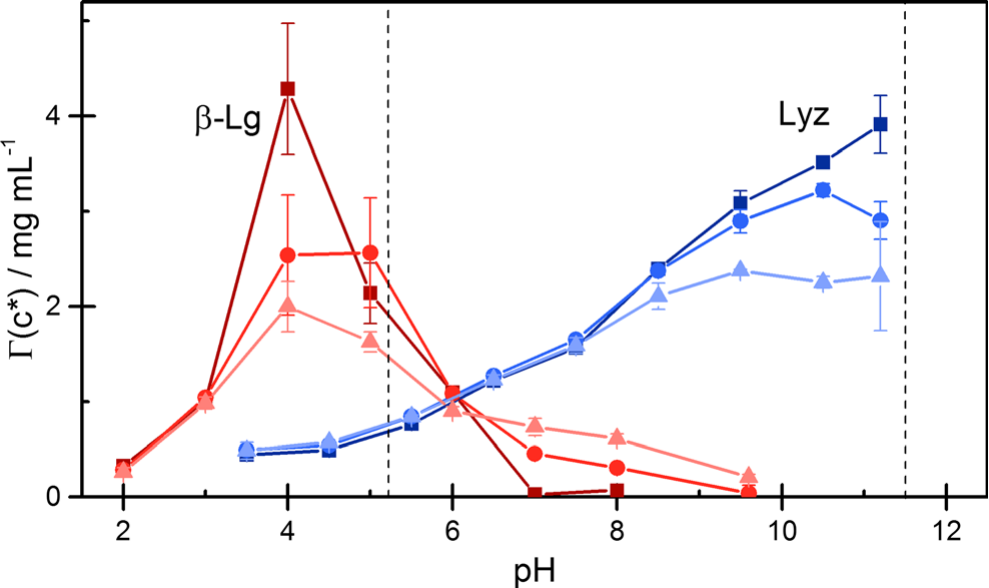
Comparison of lysozyme and ß-lactoglobulin adsorption onto silica nanoparticles: Adsorbed amount *Γ*(*c* ^*^) at the reference concentration *c* ^*^ = 2 mg/mL plotted against pH: *filled square*, no salt; *filled circle*, 25 mM NaCl; *filled triangle*, 100 mM NaCl. The isoelectric points (pI) of the proteins are indicated by *vertical dashed lines*

Because Lyz and ß-Lg have greatly different values of pI, electrostatic interactions with the negative silica surface are causing a different dependence of adsorption on pH. A comparison of the adsorption of the two proteins at the silica NPs is shown in Fig. 8, where the surface concentration *Γ*(c^*^) of adsorbed protein at a common concentration c^*^ = 2 mg/mL is plotted as a function of pH. For Lyz, where weak adsorption states play no major role, the values of *Γ*(c^*^) are similar to those of *Γ*_*m*_ at given pH and ionic strength (see Fig. 4d). In the case of ß-Lg, for which weak adsorption states are significant in the neighborhood of pI, values of *Γ*(c^*^) are higher than *Γ*_*m*_ at pH values close to pI (see Fig. 7d).

For Lyz in the absence of salt, the surface concentration *Γ*(c^*^) increases with pH in a nearly linear manner in the entire experimental pH range up to pH 11.2 ≈ pI.

At pH <7, the surface concentration is less than a complete monolayer. In this regime, added salt reduces the initial high-affinity adsorption but promotes further adsorption at higher protein concentrations (Fig. 3a). The salt-induced reduction of high-affinity adsorption can be attributed to a screening of the attractive electrostatic interaction between protein and the surface (lowering of the binding constant *K*_*S*_), and the salt-induced promotion of adsorption at higher protein concentrations can be attributed to a screening of the repulsive electrostatic interactions between protein molecules in the adsorbed layer. The interplay of these two effects causes the observed change in isotherm shape (Fig. 3a) and a weak increase in *Γ*(c^*^) with salt concentration in the region below pH 7, as shown in Fig. 8.

At pH >8 the adsorbed amount of Lyz exceeds the amount corresponding to a densely packed monolayer. In this pH regime, added salt causes a weaker increase of *Γ*(c^*^) with pH than in the absence of salt, and a maximum in *Γ*(c^*^) appears at a pH near pI. With increasing salt concentration, this maximum becomes more shallow, but we are unable to decide whether it is located at or somewhat below pI, due to the limited precision of our data and the lack of data for pH > pI. Our results do not confirm the existence of a sharp adsorption maximum at a pH < pI reported for the adsorption of Lyz on a flat silica surface in the absence of salt [24], but except for this point, our results are consistent with those reported in ref. [24]. In particular, we also find that added salt causes a decrease in the adsorbed amount in a range of pH < pI, in which the adsorbed amount exceeds one monolayer of protein molecules. This finding is surprising at first sight in view of the notion that higher ionic strength reduces the repulsive protein–protein interaction and thus enhances adsorption. Presumably, the formation of a second adsorbed protein layer in the pH region near pI involves attractive electrostatic interactions between oppositely charged patches on protein molecules in the first and second layer. An increase of ionic strength will screen these interactions and thus cause a reduction of the adsorbed amount, as it is observed for Lyz in this study. Indeed, at 100 mM NaCl, the maximum adsorption of Lyz near pI has been reduced to hardly more than one nominal monolayer (Fig. 8).

In our earlier work [2, 3], we found that pH and added salt has a pronounced influence on the protein-induced aggregation of silica NPs near the isoelectric point. In the absence of salt, large-scale aggregation occurs over a wide pH range, but the aggregates re-disperse at pH 10. Hence, at pH ≥ 10, in the absence of salt, the observed adsorbed amount represents the adsorption onto isolated (non-aggregated) silica NPs. On the other hand, in the presence of 100 mM NaCl salt, the silica-protein hetero-aggregates do not redisperse near pI [2, 3], and thus, the measured adsorbed amount represents the amount adsorbed in the confined geometry between silica particles. Presumably, part of the observed salt-induced decrease in the adsorbed amount near pH 11 is caused by this transition from adsorption onto free particles to adsorption between silica particles in the large-scale aggregates.

For ß-Lg, we could characterize the adsorption behavior for pH values on both sides of the isoelectric point (Fig. 8). In the pH regime below pI, the dependence of *Γ*(c^*^) on pH and salt concentration qualitatively resembles the behavior of Lyz, although the variation of *Γ*(c^*^) with pH is occurring in a narrow pH region due to the low value of pI. Since the silica NPs used in this study are negatively charged down to pH 2 (cf. Fig. 1a and Table 2), the similar pH and salt dependence of *Γ*(c^*^) of the two proteins at pH < pI indicates that in both cases, the behavior is dominated by electrostatic interactions. The low level of adsorption up to pH 3 indicates that in this regime, the attractive protein–surface interactions are nearly balanced by repulsive protein–protein interactions. Both of these interactions are screened by added salt, so that the adsorption level is only weakly affected by salt. The very strong increase in adsorption from pH 3 to pH 4 in the absence of salt, to values much beyond one nominal monolayer, may again be attributed to a transition from repulsive to attractive protein–protein interactions in the pH range near pI. This interpretation is supported by the salt-induced reduction of adsorption at this pH. Interestingly, the adsorbed amount at pH 5 (closest to pI) is much lower than this maximum value and less dependent on the ionic strength. In an earlier study of ß-Lg adsorption onto silica surfaces, Elofsson et al. [31] reported that the pH dependence of adsorption was caused mainly by the pH dependent variation in self-association of the protein in solution. At room temperature and pH values below 4 and above 5.2, the protein exists predominantly in form of dimers and monomers, with an increasing tendency for the dimer to dissociate into monomers at lower and higher pH, respectively. The dissociation of the dimer is the strongest in the absence of salt, due to a higher (less screened) electrostatic repulsion between the monomeric units [32]. In a narrow pH region near pH 4.6, the dimers aggregate to a larger oligomeric unit (presumably an octamer), and this secondary aggregation is enhanced by a decrease in ionic strength [31, 32]. It is tempting to attribute the very high adsorption of ß-Lg at pH 4 in the absence of salt, and its strong dependence on the ionic strength at this pH, to the adsorption of this higher oligomer. However, in this case, one would expect a high value of *Γ*(c^*^) not only at pH 4 but also at pH 5 in the absence of salt, which is not the case. In this context, we also have to consider that according to a recent report [9], the monomer–dimer association equilibrium of ß-Lg in the adsorbed state is affected by curvature of the adsorbing surface. In a study of ß-Lg adsorption at a nanoscale hydrophobic surface, it was found [9] that the association is weakened by surface curvature, to the extent that no adsorbed dimers were detectable on particles of 25 nm diameter. It would be of interest to find out if such a curvature dependence of protein association also prevails in the adsorption onto hydrophilic NPs.

The adsorption behavior of ß-Lg at pH > pI, where the protein has a negative net charge and is electrostatically repelled by the equally charged surface, confirms that adsorption of globular proteins on the “wrong side” of the isoelectric point is not limited to polyelectrolyte brushes [15–18] but can also occur on charged inorganic surfaces [21, 24]. Adsorption of ß-Lg at pH > pI may involve electrostatic interactions with the negative silica surface, either due to the persistence of positive patches at the protein surface, or due to charge regulation effects [20, 21]. Non-electrostatic contributions to the adsorption energy must also play a significant role in the adsorption of this protein on the silica NPs. At pH 7 and 8, the repulsive electrostatic protein–surface and protein–protein interaction can over-compensate this attractive non-electrostatic adsorption energy in the absence of salt, so that no adsorption occurs. In the presence of salt, the repulsive electrostatic interactions are screened and the non-electrostatic adsorption energy dominates, causing increasing adsorption with increasing salt concentration. Hence, the observed inversion of the salt dependence of the adsorption at pH > pI can be attributed to the competition of electrostatic and non-electrostatic contributions to the adsorption energy.

As a final remark, we have to point out that the adsorption of the proteins will be affected by the surface chemistry of the silica NPs. This applies particularly to the adsorption behavior at low pH. As shown in Fig. 1a, the Ludox TMA particles used in the present work have a negative zeta potential down to pH 2. In contrast, the silica NPs of our earlier work [2], which were prepared by a different route than Ludox TMA, had a zeta potential near zero below pH 4, and no adsorption of Lyz was found on these particles below pH 4. This difference in adsorption behavior at low pH can again be rationalized by electrostatic interactions as outlined above.

## 5. Conclusions

The present study has highlighted the important role of electrostatic interactions in the adsorption of the globular proteins Lyz and ß-Lg onto negatively charged silica nanoparticles. For both proteins, two adsorption regimes as a function of pH were identified for pH < pI: At low pH, the competition of attractive protein–surface interactions with the repulsive protein–protein interactions causes adsorption limited to one monolayer of protein molecules. At pH values closer to pI, repulsive interactions between protein molecules become less important and attractive protein–protein interactions resulting from oppositely charged patches on two proteins become relevant, leading to adsorption well above one monolayer of protein at low ionic strength. In the case of ß-Lg (pI ≈ 5), for which the adsorption behavior could be studied on both sides of pI, a pronounced maximum in adsorption was observed somewhat below pI in the absence of salt, and an inversion of the salt effect on the adsorption level was found in the pH region around pI. This inversion is attributed to a competition of electrostatic and non-electrostatic contributions to the adsorption energy. The role of protein association to dimers and higher oligomers appears to dominate the adsorption behavior near pI, but further work is needed to clarify details of this behavior.
